# Feeding type and microplastic particle concentration drive the ingestion of polystyrene beads in a natural zooplankton community

**DOI:** 10.1007/s10661-026-16130-2

**Published:** 2026-11-12

**Authors:** Claudia Drago, Guntram Weithoff

**Affiliations:** 1 Umweltbundesamt, 12307 Berlin, Germany; 2 Aquatic Ecology Group, University of Potsdam, 14469 Potsdam, Germany

**Keywords:** Microplastics, Zooplankton community, Ingestion, Rotifer, Copepod, Cladoceran, Concentration

## Abstract

The mismanagement of plastic waste in recent decades has led to global plastic pollution problem. Much of this plastic waste in the environment degrades into small particles, forming microplastics and increasing their abundance in ecosystems. These microplastics can be ingested by various microinvertebrates such as zooplankton. Using a microcosm experiment with a near-natural zooplankton community, we tested whether microplastics particle size and concentration affect the proportion of individuals ingesting polystyrene beads. Zooplankton were exposed to three bead sizes (1, 3, and 6 µm) at two concentrations (10^3^ and 10^6^ particles mL⁻^1^). Ingestion was assessed as the presence or absence of beads within individual organisms. As all beads sizes were within the ingestible range, the experiment was designed to evaluate size-dependent differences in ingestion probability rather than physical ingestion limit. At low concentrations, 40% of the cladocerans ingested microplastics, which was a higher percentage than copepods, rotifers and ostracods; suggesting that their unselective filter-feeding mode facilitates ingestion. At high microplastics concentrations, these were ingested by more than 70% of the organisms across all experimental groups, with only moderate differences in the proportion of individuals ingesting microplastics among rotifers, cladocerans, ostracods and copepods. These high percentages indicate that even raptorial feeders cannot avoid ingesting plastic at elevated concentrations. The size of the microplastics had no effect on their ingestibility. Overall, our near-natural experiment demonstrated that both feeding type and beads concentration significantly influenced microplastics ingestion by zooplankton.

**Supplementary Information:**

The online version contains supplementary material available at https://doi.org/10.1007/s10661-026-16130-2.

## Introduction

A global production of 430 million tons of plastic per year was estimated in 2024 (Lebreton & Andrady, 2019; Orth & Bruder, 2025). Although European production has recently decreased by approximately 10%, the global trend is continuously increasing. The overuse and mismanagement of plastic waste have led to a severe environmental challenge (Horton et al., 2017). Due to its accumulation in the environment without effective removal policies, plastic waste negatively affects macro- and microorganisms that ingest or come into contact with it (Li et al., 2024; Yang et al., 2024). Large plastics can degrade over time or break down during use and disposal (Baby et al., 2024; Eerkes-Medrano et al., 2015), resulting in secondary microplastics (MPs) of different sizes and shapes. In addition, primary MPs are intentionally produced at microscopic sizes and added to a manifold of commercial products, for example, as abrasive granules in cosmetics (Bikiaris et al., 2024; Nikiema & Asiedu, 2022). Through wastewater treatment plant effluents, stormwater runoff, and agricultural activities, these MPs end up in freshwater environments such as rivers and lakes (Haque et al., 2024; Wang et al., 2022). Within this system, MPs can partially accumulate in the sediment or be transported to marine environments and enter the ocean (He et al., 2021; Napper et al., 2023; Talbot & Chang, 2022; Wang et al., 2024). Currently, accurately quantifying and measuring the size and shape of small plastics (1–50 µm) in complex environmental samples, such as freshwater or sediment, especially for the size range <20 µm, remains a challenge. Regardless of these limitations, the concentration of the smallest fraction is estimated to increase exponentially compared to actual measurements (Gola et al., 2021; Lindeque et al., 2020; Mai et al., 2018; Petersen & Hubbart, 2021; Yang et al., 2023). Once in the environment, MPs interact with bacteria, detritus and algae undergoing processes such as heteroaggregation (Parrella et al., 2024; Su et al., 2025). This aggregation can alter the fate of MPs, changing their sinking behavior, transportation and ecological effects (Leiser et al., 2020; Wang et al., 2021). The aggregation of MPs with biofilm or algae may also be ingested by consumers (such as zooplankton, zoobenthos and fish) (Drago et al., 2020; Yu et al., 2020). Zooplankton are a basic component of aquatic food webs, serving as a primary food source for fish and larger predatory organisms and as consumers of phytoplankton. The feeding mechanism of zooplankton, specifically filter-feeding versus raptorial strategies, particle size and morphology as well as the encounter rate, are key factors dictating ingestion rates (Cole et al., 2013). In particular, the encounter rate in raptorial zooplankton organisms such as copepods depends not only on the concentration of MP particles but also on swimming speed and environmental conditions as turbulent or calm surface water (Sanvicente-Añorve et al., 2023). In ecological and limnological studies, quantifying ingestion rates is essential for modelling population dynamics and assessing competitive resource interactions within zooplankton communities.

Upon ingestion, MPs particles can cause mechanical damage to the digestive system of zooplankton, as they can remain in the digestive system for several days (Cole et al., 2013). For example, in copepods, the ingestion of MPs particles of a small size category (1–8 µm) can reduce the ingestion of similarly sized algae (Cole et al., 2013) or interfere with the ingestion of algal food as shown in the study of Galir et al., 2025. Furthermore, rotifers display a shortened lifespan, diminished population growth rate, and impaired reproduction when exposed to MPs particles (Drago & Weithoff, 2021; Pawlak et al., 2022; Sun et al., 2019). These organisms can also convert ingested MPs into nanosized particles with their chitin mastax, contributing to the generation of nanoplastics in surface waters (Zhao et al., 2024). In cladocerans, the ingestion of smaller MPs fraction (1.5 µm) leads to a higher mortality and may impair mobility affecting biological interaction (Galir et al., 2025).

To date, numerous laboratory studies have documented the ingestion of MPs by zooplankton using controlled, single-species exposure (Botterell et al., 2019). However, few studies have investigated MPs uptake within natural communities. For instance, using a size-fractionation approach, da Silva et al., 2022 demonstrated that within a river floodplain plankton community, smaller MPs were preferentially ingested over larger particles, with protists contributing significantly to overall MPs consumption. Supporting this finding, a microcosm study involving rotifers, cladocerans, and copepods revealed that the smaller MPs fraction (1–5 µm) was ingested by the majority of the groups compared to the largest fraction (approximately 10–50 µm) (Galir et al., 2025). Fueser et al., 2020 evaluated MPs ingestion in a natural microbenthic community and revealed a direct correlation between nematode feeding modes and MPs uptake. Multispecies community experiments allow for direct inter-species comparisons, enabling the identification of which taxonomic groups are most susceptible to MP ingestion based on their feeding mode and the size range of MPs. This identifies organisms vulnerable to adverse effects or those likely to facilitate trophic transfer to higher consumers (Jiménez-Contreras et al., 2026; Setälä et al., 2014, 2016).

The primary objective of this study was to screen different zooplankton groups, including rotifers, cladocerans, copepods, and ostracods, for the presence of ingested MPs and to assess whether the proportion of individuals containing MPs differed among three beads sizes (1, 3, and 6 µm) and two exposure concentrations (10^3^ and 10^6^ particles mL⁻^1^) over a short exposure time of 24 h. The higher MPs concentration enable to increase the encounter rate in a 24 h exposure time and avoid excessive predation and egestion of MPs. By determining the proportion of organisms in which MPs were detected, this study provides insights into which zooplankton taxa were more likely to ingest the three tested MPs sizes and how MPs occurrence varied between the two exposure concentrations.

## Material and methods

### Microplastics

As MPs particles, we used standard fluorescently labelled polystyrene microbeads of three different diameters: 1.03, 3.06, and 5.73 µm (Polysciences, Inc. Fluoresbrite YG Polystyrene Microspheres, USA). For convenience, we refer to them as 1, 3 and 6 PS. A stock solution was prepared using deionized Milli-Q water under sterile conditions to minimize bacterial growth. To keep the MPs as singular particles, each stock solution was sonicated for 30 min and mixed using a vortex mixer prior to use.

### Experimental procedure

Approximately 50 L of water from an urban lake (Heiliger See, Potsdam, Germany, 52° 24′ 40″N, 13° 04′ 17″E) was collected to get the zooplankton community as much similar as possible to natural conditions. This community was concentrated by sieving through a 30 µm mesh and acclimatized to laboratory conditions overnight in particle-free lake water filtered with a 0.8 µm mesh, and a mixed algal food (*Monoraphidium minutum* with a concentration of 10^6^ cell mL^−1^ and *Chlamydomonas noctigama* with a concentration of 10^5^ cell mL^−1^) was provided. These two algal species are commonly used to feed laboratory cultures of rotifers and cladocerans because of their good food quality (Piepho et al., 2012; Wojcik et al., 2025).

Prior to the experiment, 200 mL of water containing zooplankton was sieved again through a 30 µm mesh to remove algal food, resuspended with 200 mL of particle-free lake water, and gently mixed to maintain a random distribution. The organism concentration was determined by counting individuals under a stereomicroscope in a Petri dish and adjusted to the desired concentration with particle-free lake water. For each replicate, 10 mL of this stock with a concentration of 15 ± 5 organisms mL^−1^ was transferred into glass jars, and MPs were added at the target concentration. To reach an equilibrium between the ingestion and egestion time of each group of organisms and avoid the re-ingestion of the same beads, the experiment was run for 24 h in the dark. Glass beakers containing different organisms were placed on a rocker shaker (10 movements per minute) to slow down the sedimentation of larger MPs. We used two concentrations of MPs for each size: 10^3^ and 10^6^ particles mL^−1^ and each size and concentration treatment were performed in triplicate, with in total 18 experimental glass beakers. Both MPs concentrations utilized: 10^3^ and 10^6^ particles mL^−1^ are referred later on respectively as “low” and “high” MPs concentration. These two concentrations exceeded the typical MPs concentrations in the environment; nevertheless, they were chosen as a compromise between possible re-ingestion of MPs (Bermúdez et al., 2025), predation among groups of organisms (Jiménez-Contreras et al., 2026), and ensuring a high encounter rate of MPs (Ogonowski et al., 2016). However, low MPs concentration (10^3^ mL^−1^) is intended to mimic a pollution pulse event.

This experimental design allowed us to investigate particle availability and assess whether the proportion of zooplankton individuals ingesting MPs differed among beads sizes and zooplankton groups.

When the experiment was terminated, the organisms were fixed with 90% ethanol (Parry et al., 2024) and preserved in the refrigerator at 8 °C until microscopic inspection. The samples were preserved for 1 month. Choosing ethanol, we avoid any possible autofluorescence and we consider a more environmentally friendly approach than formalin. Before inspection the samples were washed with copious sterile water. For each zooplankton individual, the presence or absence of fluorescent polystyrene beads was visually assessed under the fluorescent microscope (Zeiss, Axioskop, Germany). Bead size was assigned according to the corresponding experimental treatment (1, 3, or 6 µm). Individuals in which at least one bead was detected within the body were classified as positive for MP ingestion. The number of beads per individual was not quantified. For each treatment, the proportion of individuals positive for MPs ingestion was calculated. Organisms without MPs had either not ingested MPs or had already egested it. Zooplankton were identified to the genus level, except for copepods, which were classified to the order level (Calanoida and Cyclopoida) and developmental stage (Nauplii), and ostracods, which were classified to the class level (Ostracoda) (Bledzki & Rybak, 2016). When necessary, Calcofluor White Stain (Sigma-Aldrich Chemie GmbH, Germany) was used to facilitate rotifer identification (Koste & Voigt, 1978; Segers, 2007) by staining the trophi while preserving the organisms (Parry et al., 2024).

### Statistical analysis

The percentage of organisms that ingested beads was calculated for each replicate. Kruskal–Wallis test was first applied to investigate differences in the percentage of organisms with ingested beads among the beads size (1-, 3-, 6 µm) and between the concentrations (high and low). The difference among the groups was investigated by the Kruskal–Wallis test and when significant, the Wilcoxon-Mann–Whitney test was performed with Bonferroni correction. The same analysis was performed separately for the genera *Keratella* and *Trichocerca* and for the Calanoida and Cyclopoida classes. The analysis was performed using RStudio (Posit team, 2023).

## Results

In total, 1740 individual organisms were examined (see Table S1 for total number of organisms). Seventeen genera were identified for rotifers and cladocerans, and copepods were divided into Calanoida, Cyclopoida, and nauplii that were not assigned to a taxonomic group (Table 1).

**Table 1:** Zooplankton community composition with feeding mode and feeding size range preference

Group	Genus/class	Feeding mode	Feeding size range	Reference
Rotifera	***Keratella***	Filter feeders/polyphagous	~1–2 µm	(Gilbert, 2022; Ronneberger, 1998)
	*Keratella quadrata*	Filter feeders/polyphagous	~1–2 µm	(Ronneberger, 1998)
	***Trichocerca***	Raptorial/macropahgous	~0.5 μm and filamentous algae	(Gilbert, 2022)
	*Lecane*	Detritivore/microphagous	~1–10 µm	(Arcifa et al., 2020; Gilbert, 2022)
	*Lepadella*	Bacterivory/microphagous	~1–10 µm	(Gilbert, 2022)
	*Colurella*	Grazing feeders	~1–10 µm	(Arcifa et al., 2020)
	*Sincheta*	Omnivourus/macrophagous	~0.5–50 µm	(Gilbert, 2022)
	*Cephalodella*	Raptorial/omnivourus	~2–10 µm	(Weithoff, 2005)
	*Kellicottia*	Filter feeders/polyphagous	~2–10 µm	(Gilbert, 2022)
	*Monommata*	Raptorial	Not available	(Obertegger et al., 2011)
	*Polyarthra*	Raptorial/omnivourus	Nano algae and 20–50 µm	(Gilbert, 2022; Liang et al., 2022)
	*Bdelloids*	Filter feeders/microphagous	~0.5–1 µm	(He et al., 2022)
	*Trichotria*	Filter feeders/microphagous	~0.5–2 µm	(Rodríguez et al., 2013)
Cladocera	*Alona*	Filter feeders	~1–40 µm	(Downing, 1981)
	*Alonella*	Filter feeders	Not available	(Adamczuk, 2013)
	*Bosmina*	Filter feeders	~3–30 µm	(Bleiwas & Stokes, 1985; Bogdan & Gilbert, 1982)
	*Pleuroxus*	Filter feeders	Not available	(Yuslan et al., 2024)
	***Ceriodaphnia***	Filter feeders	~3–15 µm	(Ganf & Shiel, 1985)
Copepoda	*Calanoida*	Filter feeders	<20 µm and >20 µm	(Cole et al., 2015; Coppock et al., 2019)
	***Cyclopoida***	Raptorials	~0.5–300 µm (10% larger than its body size)	(Saiz et al., 2014)
	**Nauplii**	Filter feeders	~2–15 µm	(Helenius & Saiz, 2017)
Ostracoda	/	Detritivore	Unspecific	(Schmit et al., 2007)

In bold the most abundant organism per group found in the experiment

**Fig. 1:**
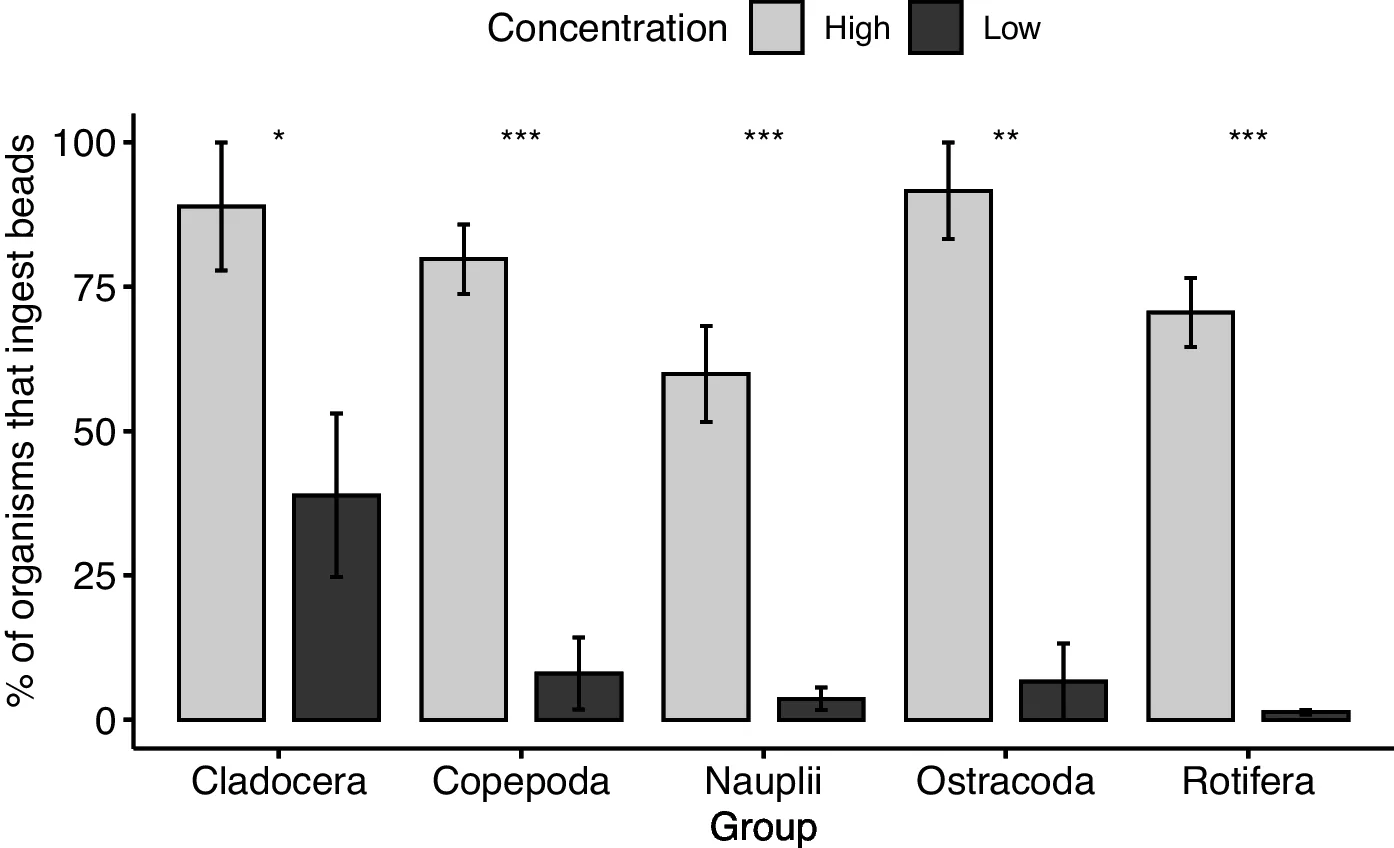
Percentage of organisms that ingested MPs (mean ± SE). The sizes of the three beads were pooled, and the stars represent significant differences between the high and low concentrations of beads. *Keratella* spp. and *Trichocerca* spp. are not included since they were analyzed separately in Fig. 3. Statistical significance: *p* < 0.05 (*), *p* < 0.01 (**)*, p* < 0.001 (***)

Polystyrene beads of all three sizes were ingested by all groups at high concentration (Fig. 1 and Table S2 for the percentage and SE). The percentage of organisms having ingested and kept beads was similar among the different size treatments (Kruskal–Wallis test: *p* > 0.05, Table S2 for percentage and SE and Table S3 for statistical significance). The results of the three MPs sizes were pooled together to assess the influence of the concentration of MPs on the percentage of ingestion, independently from the size. The number of organisms that had ingested MPs was higher (see Table 2) at high beads concentrations (Kruskal–Wallis test: Cladocera *p* < 0.05; Copepoda *p* < 0.001; Nauplii *p* < 0.001; Ostracoda *p* < 0.01; Rotifera *p* < 0.001; see Table S3 for statistical significance).

**Table 2:** Percentage of organisms that ingested beads at high and low microplastics concentrations

Zooplankton group	High concentration: ingestion (%), mean ± SE	*N* individuals	Low concentration: ingestion (%), mean ± SE	*N* individuals
***Keratella***	68.4 ± 7.5	444	0.4 ± 0.2	458
***Trichocerca***	79.5 ± 2.9	100	1.7 ± 0.9	111
**Calanoida**	83.3 ± 16.7	14	16.7 ± 16.7	7
**Cyclopoida**	79.2 ± 15.0	55	4.2 ± 4.2	26
**Nauplii**	59.2 ± 10.5	111	3.4 ± 2.4	126
Rotifera*	61.3 ± 4.7	112	7.1 ± 2.3	95
**Ostracoda**	93.9 ± 6.1	20	8.3 ± 8.3	10
**Cladocera**	95.2 ± 4.8	30	52.6 ± 12.1	21

Values are presented as mean ± SE of three replicate microcosms after pooling the three beads sizes (1, 3, and 6 µm). *N* represents the total number of individuals examined across the three beads sizes and three replicates.*Rotifera excluded *Keratella* and *Trichocerca* sp.

The percentage of organisms that had ingested MPs was not different among the groups (Kruskal–Wallis test: *p* > 0.05; see Table S4 for statistical significance).

### Rotifera

**Fig. 2:**
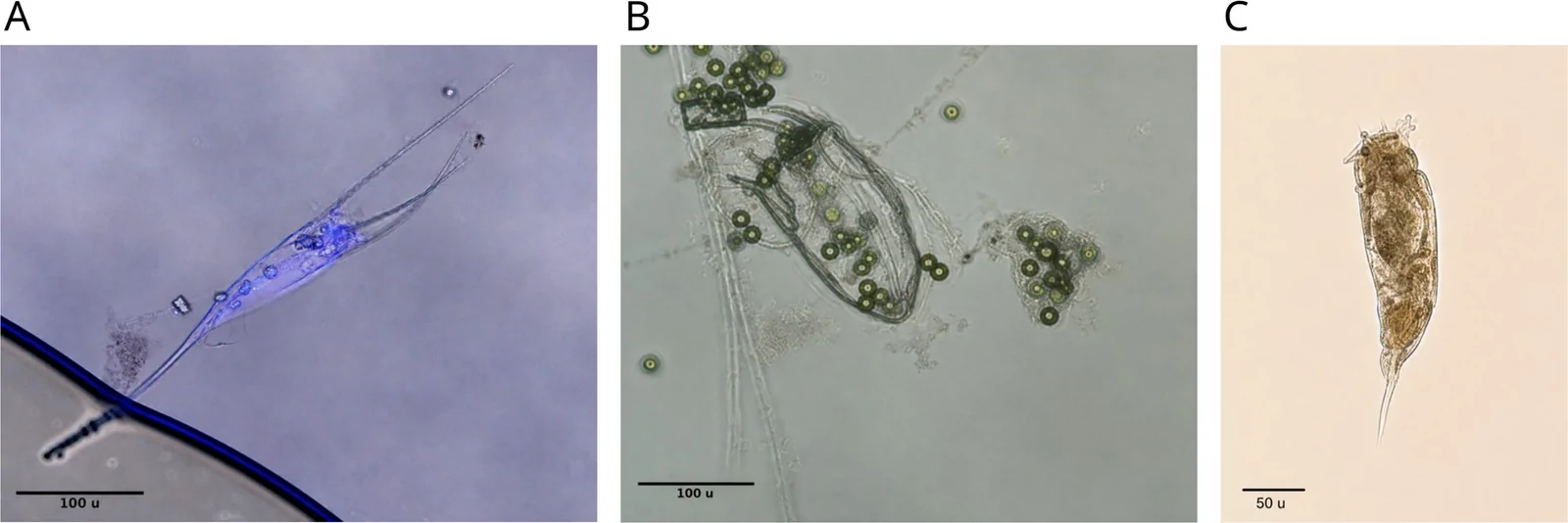
Rotifers identified in the experimental sample (**A** *Kellicottia* spp. under fluorescent UV light; **B** *Keratella* spp. with ingested 3 μm PS beads; **C** *Trichocerca* spp. under bright field)

In total, 1320 individual rotifers from eleven genera within the subclass Monogononta and one genus of Bdelloids were identified in our experiment; the abundance varied from more than 100 specimens (*Keratella* sp.) to one specimen (*Kellicottia* sp.) per sample. The majority of rotifers ingested all three sizes of beads (Fig. 2A, B C; Table S1).

**Fig. 3:**
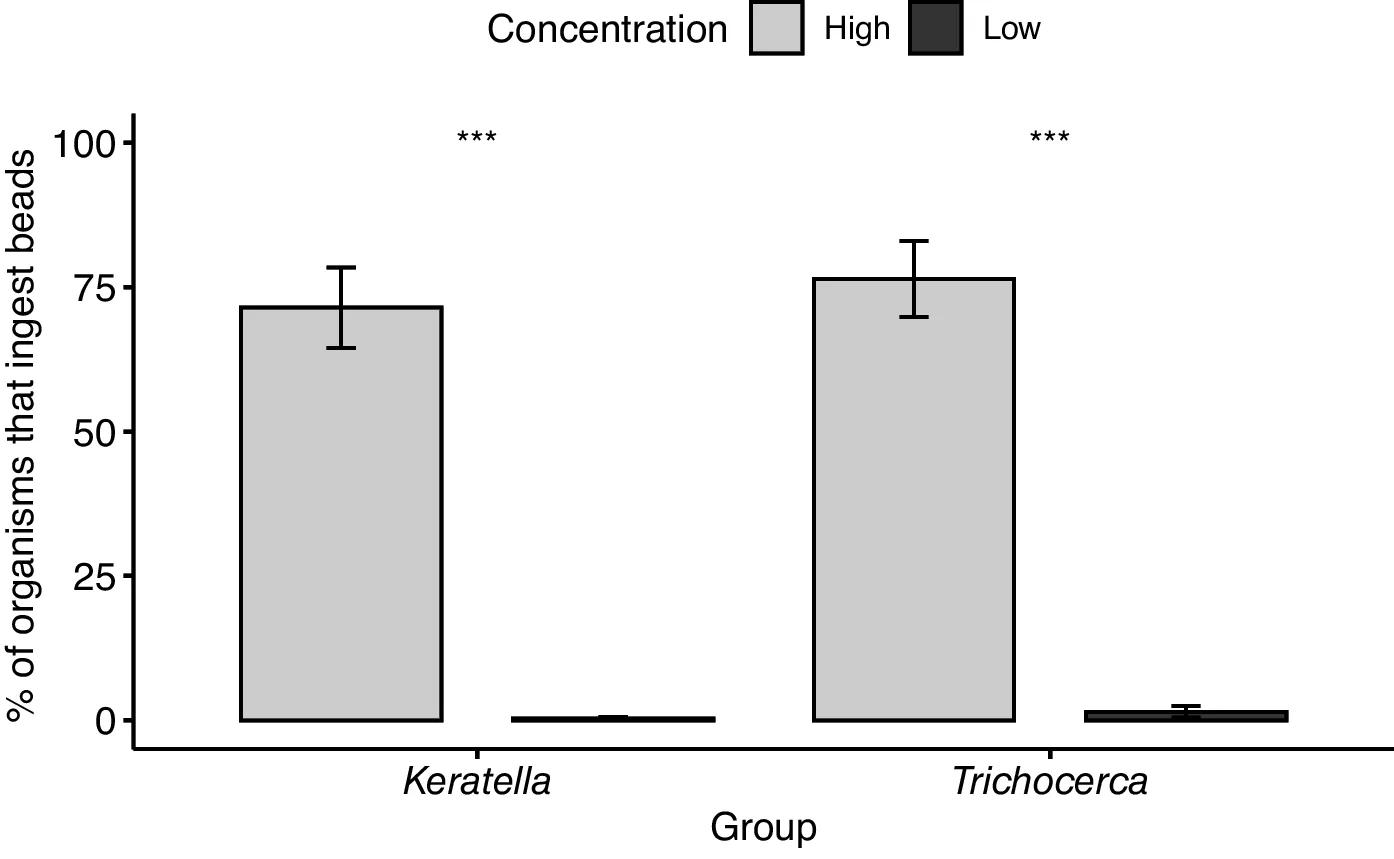
Percentage of organisms (**A** *Keratella* sp. and *Trichocerca* sp.) that ingested MPs beads (mean ± SE). The three bead sizes were pooled, and the stars represent significant differences between high and low MPs concentrations. Statistical significance: *p* < 0.05 (*),* p* < *0.01 *(**), *p* < *0.001 *(***)

The ingestion of the two most abundant genera, *Trichocerca* and *Keratella*, was analyzed separately (Fig. 3), as their high abundance could have disproportionately influenced the overall results for the Rotifera group.

We found a higher percentage of organisms that ingested beads at higher concentrations (see Table 2), but no difference among the sizes. The percentages of *Keratella* and *Trichocerca* that ingested MPs did not differ from each other at different sizes (see Tables S5 and S6 for statistical significance).

*Cephalodella* sp. ingested all three MP sizes at high concentrations, as did *Trichocerca*, *Keratella,* and *Syncheta* sp. Some genera were less abundant than others, for example *Kellicottia* sp., *Lecane* sp., and *Lepadella* sp*,* and they had ingested or kept the beads selectively; at high MPs concentrations, in *Colurella* sp. only 1 and 3 µm beads were found or in *Trichotria* sp. only 1 and 6 µm beads (see Table. S1 for ingestion of organisms per size).

### Cladocera

**Fig. 4:**
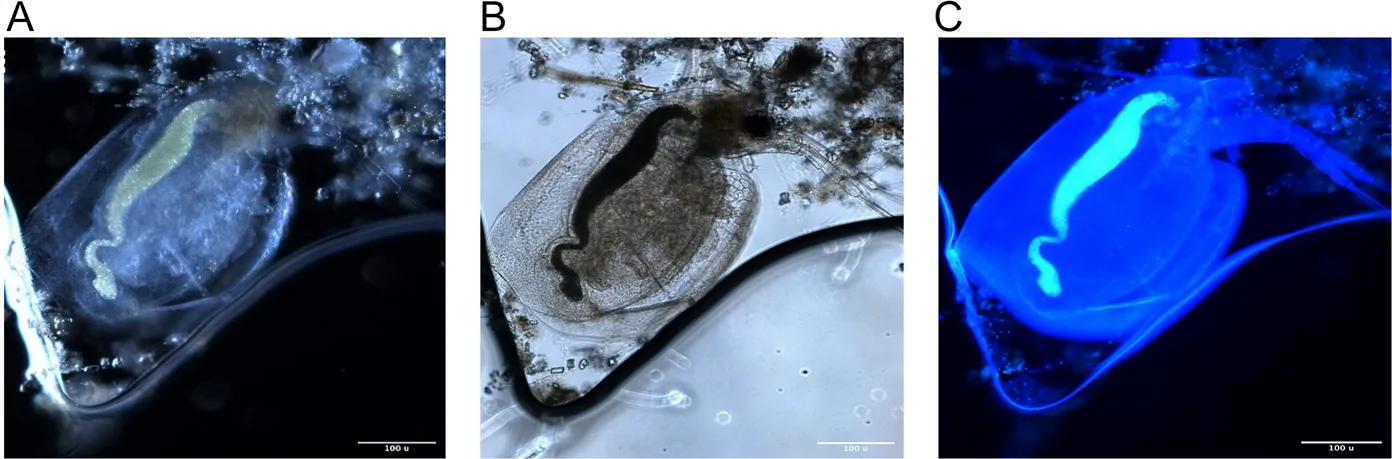
The cladocerans identified in the experimental sample, *Ceriodaphnia* sp. (**A** under dark field light; **B** under bright field light; **C** under UV fluorescent light). The organism's gut appeared to be filled with 3 μm beads

In total, 51 individuals from five genera were identified, and their abundance varied among the MPs size treatments. Among all groups, Cladocera exhibited the highest percentage of individuals that had ingested and kept beads at low concentrations (Fig. 1 and Table 2). In particular, *Ceriodaphnia* was present in every treatment and ingested all three bead sizes as well as *Alonella*. Only a few specimens of *Alona*, *Bosmina* and *Pleuroxus* were found, and they had variable ingestion. The most abundant genus was *Ceriodaphnia,* with 33 individuals, the majority of which ingested and kept the beads at high and low bead concentrations (Fig. 4; see Table. S1 for the total number of organisms).

### Copepoda

The copepods were classified as Calanoida, Cyclopoida, and nauplii, with 21 Calanoida, 81 Cyclopoida, and 237 nauplii found. Nauplii are not included as their high abundance could have disproportionately influenced the overall results for the Copepoda group.

**Fig. 5:**
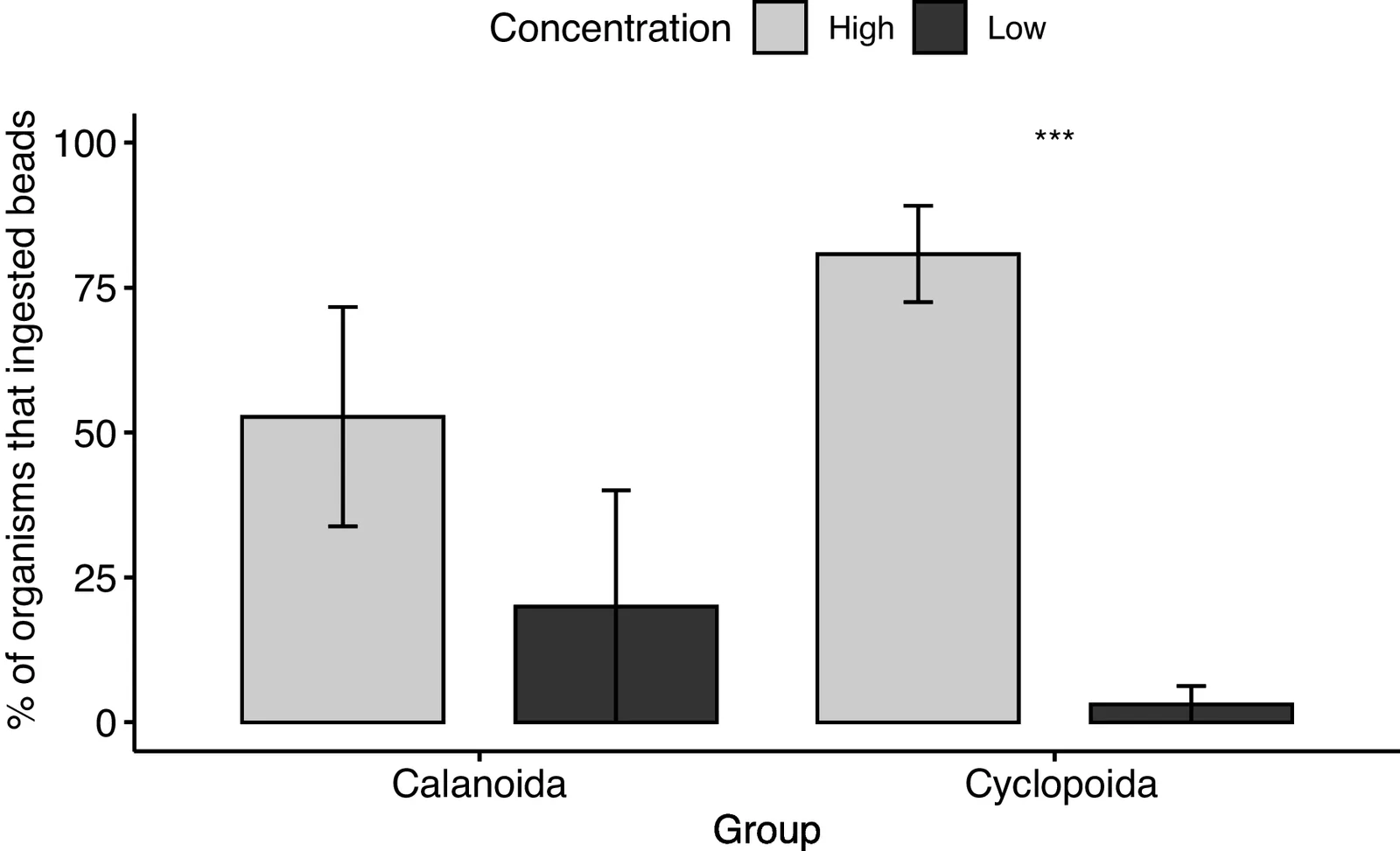
Percentage of organisms (Calanoida and *Cyclopoida*) that ingested the beads (mean ± SE). The three bead sizes were pooled, and the stars represent significant differences between high and low MPs concentrations. Statistical significance: *p* < 0.05 (*), *p* < *0.01 *(**), *p* < *0.001 *(***)

There was no difference between the two classes in the percentage of organisms that ingested and kept beads, for calanoida there was no difference even for high and low beads concentrations (Kruskal–Wallis test; *p* = 0.085; see Table S8 for statistical significance; Fig. 5). The percentage of organisms that ingested the beads decreased significantly at low beads concentrations for cyclopoids (Kruskal–Wallis test; *p* < 0.001; see Table S7 for statistical significance).

### Ostracoda

A total of 30 organisms were identified in the ostracod group across the entire experimental setup. These organisms were capable of ingesting MPs beads of all three sizes at both high and low concentrations (Table 2). However, the percentage of organisms that ingested the beads decreased significantly at lower concentrations (Kruskal–Wallis, *p* < 0.01; see Table S7 for statistical significance).

## Discussion

The aim of this study was to investigate whether MPs concentration, together with the feeding behavior and size-dependent ingestion of different zooplankton groups, influenced MPs bioavailability and the proportion of organisms ingesting MPs. A higher proportion of organisms ingested MPs when exposed to the extremely high MPs concentration, whereas a lower proportion ingested MPs at the lower concentration. These findings suggest that, at higher MPs concentrations, beads may become more difficult for organisms to avoid, whereas at lower concentrations, the feeding mode of each organism may have a greater influence on MPs ingestion. In particular, some groups, such as raptorial rotifers and copepods, may be more capable of avoiding or rapidly egesting MPs. Similar patterns have also been reported for benthic communities, as shown by Fueser et al., (2020), and for planktonic communities, as reported by Setälä et al., (2014, 2016). In these studies, a higher proportion of copepods and rotifers ingested MPs at higher concentrations.

### Beads sizes

Beads of all three sizes (1, 3, and 6 µm) were ingested by all groups and nearly all organisms. Although each group or species has its own preferred food size range, the tested sizes fell within the ingestible range for all groups. In fact, several rotifers and cladocerans feed efficiently on 1, 3, and 6 µm beads (Drago et al., 2020; Jaikumar et al., 2019; Kirk, 1991; Pagano, 2008; Pawlak et al., 2022). Field studies and laboratory experiments have shown that several calanoid species are capable of ingesting particles across a very broad size range: in the field from 21 to 280 µm, and in laboratory conditions from 0.05, 0.5, to 6 µm beads (Cole et al., 2015; Coppock et al., 2019; Yu et al., 2020). A study (da Silva et al., 2022), which applied a similar experimental setup, demonstrated that 0.5, 1, and 3 µm MPs were consumed by all zooplankton taxa (from protists to cladocerans) in a natural zooplankton community from a river floodplain. Our results are also in accordance with a mesocosm study in which a community formed by rotifers, cladocerans, and copepods was exposed to different size ranges of MPs; in this experiment, the smallest size of MPs (1–5 µm) was ingested by the majority of the rotifers, cladocerans, and copepods (Galir et al., 2025). In general, for filter feeders, the minimum ingestible particle size is determined by the mesh size of the filtering apparatus, whereas the maximum size is constrained by the morphology of the mouthparts and, in cladocerans, the carapace gap (Scherer et al., 2017). In the environment, MPs can aggregate by themselves and with bacteria and algae. This aggregation alters the availability of the initial size range, while the resulting biological coating (or eco-corona) may attract consumers (Vroom et al., 2017). This likely explains why no significant differences were observed among the three sizes. However, for rotifers some degree of size-related selectivity in ingestion may still occur, as reported for several taxa with different feeding morphologies. In particular, smaller rotifers such as *Keratella* and *Cephalodella* are known to readily ingest very small particles (e.g. 1–2 µm), while showing comparatively lower efficiency in ingesting larger particles such as 6 µm (Gilbert, 2022). For other larger rotifer taxa, such as *Brachionus* spp., particles in the 3–6 µm range are generally ingested more efficiently, whereas uptake of 1 µm particles may be less effective (Starkweather, 1980; Starkweather et al., 1979). Therefore, compared to the other zooplankton groups, rotifers may exhibit a stronger potential for selective ingestion based on particle size, even within the size range tested.

Because of the uncountable number of microplastic particles within some individuals, we did not quantify the consumption rates but recorded only the presence or absence of MPs beads in each organism. Therefore, our study provides qualitative evidence of the MPs beads ingestion rather than a quantitative estimation of ingestion rate per organisms in a natural environment, where MPs concentration and characteristic as well as food condition may differ from this experimental setup. The aim of this study was to investigate if the percentage of organisms that ingested the beads would change accordingly to the size range selected (1, 3, and 6 µm) and to identify preference among the groups. Since no substantial differences in the percentage of organisms ingesting a specific size of beads was observed, we pooled the results across the three bead sizes to investigate the differences among the groups in relation to the concentration of beads.

### Beads concentration

The two concentrations used in this study were extremely high compared to environmental MPs concentration. In freshwater environments, the mean concentration of MPs ranges from approximately 0.5 MP L^−1^ to 100 MP L^−1^ in urban rivers, and the smallest size fraction considered is 25–50 µm in size (Mandal et al., 2025; Semmouri et al., 2023). The analysis of the smallest fraction (1–10 µm) is still a challenge and it is underestimated due to analytical limitations. In addition, extrapolating data on the actual concentration of MPs in freshwater environments indicates that the smallest fraction is four orders of magnitude underestimated (D’Avignon et al., 2022; Molazadeh et al., 2023). The concentrations used here are a compromise considering the short exposure time and ensuring a higher encounter rate, providing a screening of organisms more susceptible to ingest MPs. Moreover, the low concentration of MPs used in this study mimics a single pollution pulse event, such as floods, stormwater runoff, and rain events, which can increase the MPs load by approximately 50 times (Forrest et al., 2022).

In this study, more than 70% of the organisms ingested and kept MPs at high concentration, whereas this proportion declined to approximately 40% at lower concentrations. The highest percentage of ingestion was observed in ostracods, followed by cladocerans, copepods and rotifers.

Planktonic filter feeders are generally more prone to unintentionally ingest non-food particles from the water; however, at low concentrations, some groups, such as rotifers and copepods, appear more selective and can avoid MPs ingestion. Observational studies in the field have documented the presence of MPs in different zooplankton groups, including copepods, cladocerans, and rotifers, demonstrating that MPs ingestion can occur under natural conditions and potentially contribute to trophic transfer through aquatic food webs (Jiménez-Contreras et al., 2026). A study on a benthic community (Fueser et al., 2020) reported similar results for copepods and chironomid larvae, with high ingestion percentages at elevated bead concentrations. In a coastal brackish environment (Baltic Sea), nauplii, cladocerans, and rotifers ingested MPs at concentrations of 10^4^ beads ml⁻^1^, whereas only a few rotifers ingested MPs at a lower concentration of 10^3^ beads ml⁻^1^, consistent with our findings (Setälä et al., 2014, 2016). Increasing beads concentration leads to higher encounter rates and consumption (Scherer et al., 2017), thereby increasing the proportion of organisms that ingest MPs, as observed in our study.

At low bead concentrations, only cladocerans exhibited a percentage of ingestion above 40% among the tested groups, with even higher percentage at higher concentrations. As filter feeders, cladocerans use specialized filtering structures to sieve and retain suspended particles, making them particularly prone to MPs ingestion because of their largely unselective uptake of seston (Scherer et al., 2018). The majority of the cladocerans ingested all three sizes of MPs, and their guts appeared completely full of particles. This high particle burden could also lead to increased mortality, as documented in other studies (Galir et al., 2025). In contrast, copepods employ both filtering (calanoids) and raptorial (cyclopoids) feeding strategies, and both groups are capable of particle manipulation, resulting in high food selectivity. However, purely filter-feeding copepods may not be able to distinguish small particles or may indirectly ingest MPs, particularly at high bead concentrations (Hansen et al., 1994).

Early life stages of copepods exploit resources according to their body size, especially with respect to the minimum particle size (Hansen et al., 1994). Raptorial feeders (e.g., cyclopoid copepods and some rotifers) can selectively target specific algal sizes or phytoflagellates, and high concentrations of smaller particles do not necessarily interfere with the ingestion of their preferred food (Bogdan & Gilbert, 1982, 1984). Studies on *Keratella* sp. have indicated that the morphology of the feeding apparatus (corona and mastax) facilitates the collection of particles of various shapes and sizes, including bacteria and detritus (Bogdan & Gilbert, 1982). *Keratella* sp. are also considered polyphagous rotifers, capable of ingesting algae ranging from 1 to 20 µm (Bogdan & Gilbert, 1984; Gilbert, 2022). For example, *Trichocerca* sp. feed on small suspended particles and filamentous algae (May et al., 2001), but can also exhibit raptorial behavior, such as feeding on the eggs of other rotifers (Gilbert, 2022).

Our results demonstrate that these two rotifer genera exhibit low ingestion of MPs at low concentrations. In fact, *Keratella* sp. is well known for its selective ingestion size range, with a preference for smaller particles (1–2 µm) (DeMott, 1986; Ooms-Wilms, 1997), whereas *Trichocerca* sp., due to its specialized virgate trophi, exhibits a more raptorial feeding strategy, grasping and piercing its food (Oh et al., 2017). However, studies investigating ingestion preferences in *Trichocerca* sp. are scarce, as are studies on microplastic ingestion by these two genera. A similar pattern was observed in a mesocosm experiment, where the ingestion of MPs in copepods was lower than the ingestion in bigger zooplankton as Daphnia (Yıldız et al., 2022), and in a study on ostracods, which feed on algae and detritus and exploit resources at the sediment–water interface (Thorp & Covich, 2009).

### Further implications

Due to technical limitations, the quantification of MPs in the environment is likely to be underestimated. Nevertheless, projections indicate that environmental concentrations will increase in the coming decades (Lindeque et al., 2020; Petersen & Hubbart, 2021). Therefore, substantial efforts are required to reduce the input of plastics into ecosystems (Borrelle et al., 2020).

In marine environments, plastics continue to degrade over time, leading to an increase in particle abundance as their size decreases (Barboza & Gimenez, 2015; Koelmans et al., 2022). As concentrations increase, a growing number of species may become susceptible to MPs ingestion. In our experiments, high concentration of MPs lead to a higher percentage of organism ingesting it, instead at lower concentration the organism could avoid the ingestion, especially the raptorial one. Only a few studies have investigated the long-term effects of MPs exposure on freshwater communities. For example, one study on micro- and meiobenthos reported slight changes in community structure (Rauchschwalbe et al., 2022). Over longer timescales (15 months), the abundance of oligochaetes was affected by MPs, whereas no effects were observed over shorter periods (3 months) (Redondo-Hasselerharm et al., 2020; Stanković et al., 2021). The zooplankton community in our microcosms consisted of species from different trophic levels; therefore, in some cases, carnivores may have ingested prey that had already taken up MPs. As this reflects natural trophic interactions, we did not consider it an experimental artifact. Since herbivorous zooplankton constitute a fundamental food source for invertebrate predators and fish, ingested MPs can be transferred to higher trophic levels, contributing to a higher exposure risk for the “predator” of the prey contaminated with MPs and potentially affect these organisms (DeMott, 1986; Sanvicente-Añorve et al., 2023; Setälä et al., 2014). This effect becomes even more relevant when additives or adsorbed chemicals associated with MPs (Hartmann et al., 2017; Zimmermann et al., 2020) are transferred to higher-trophic levels.

## Conclusion

MPs are pervasive pollutant, accumulating in the environment, especially in the aquatic environment. This long-term exposure could lead to community changes. MPs ingestion in planktonic organisms is influenced by feeding mode and concentration, implying species-specificity effect on community level. Our experiment demonstrated that a high proportion of planktonic organisms ingested small MPs when exposed to extremely high concentrations. At lower concentrations, mimicking a pollution pulse event, only a small proportion of organisms ingest MPs, largely depending on their feeding mode. These differences in ingestion make species composition particularly relevant for the potential trophic transfer of MPs within the food web. With increasing concentrations of MPs in the environment, greater efforts are required to understand and mitigate their potential impacts. These findings highlight the importance of testing different species compositions and potential trophic transfer pathways that could depend specifically on the feeding mode of the organisms taken into account and on the concentration of MPs.

Amplifying the microcosm to a mesocosm, adding food resources and sediment, and using a higher taxonomic resolution could lead to a more ecological approach, revealing more insights into species-specificity, food-bead interactions, and potential trophic transfer. Future studies should also include the quantification of MPs consumed, either directly or indirectly using fluorescence-based approaches, to better assess MPs ingestion. Thus, studies are needed to determine how prolonged exposure and different types of MPs affect population dynamics and community structure in planktonic systems composed of diverse zooplankton groups.

## Supplementary Information

Below is the link to the electronic supplementary material.

Supplementary file1 (DOCX 33.7 KB)

Supplementary file2 (XLSX 57.9 KB)

## Data Availability

All data supporting the findings of this study are available within the paper and its Supplementary Information.
