# Supplementary material for: Feeding type and microplastic particle concentration drive the ingestion of polystyrene beads in a natural zooplankton community
Source: Environ Monit Assess. 2026 Nov 12;198(12):1300. doi: 10.1007/s10661-026-16130-2 (PMC13759585; doi:10.1007/s10661-026-16130-2)
Supplement: Supplementary file 1 [file 10661_2026_16130_MOESM1_ESM.docx]

“Feeding type and microplastic concentration drives the ingestion of polystyrene beads in a natural zooplankton community.”

Claudia Drago^1,2^*, Guntram Weithoff ^2^*

^1^Umweltbundesamt, 12307 Berlin, Germany

^2^Aquatic Ecology Group, University of Potsdam, 14469 Potsdam, Germany

*Corresponding author(s). E-mail(s): [claudra90@gmail.com](mailto:claudra90@gmail.com), weithoff@uni-potsdam.de

**Additional Information for MPs beads.**

The beads used in the experiment shared the same characteristics, as they were purchased at the same time from the same provider. The characteristics of each bead size were as follows:

- **1 µm:** 4.55 × 10^10^ particles/mL; coefficient of variation (CV) = 3%; excitation maximum = 441 nm; emission maximum = 486 nm.
- **3 µm:** 1.68 × 10⁹ particles/mL; coefficient of variation (CV) = 5%; excitation maximum = 441 nm; emission maximum = 486 nm.
- **6 µm:** 2.10 × 10⁸ particles/mL; coefficient of variation (CV) = 10%; excitation maximum = 441 nm; emission maximum = 486 nm.

We prepared intermediate stock solutions at 10⁷ particles mL⁻¹ and 10⁴ particles mL⁻¹, corresponding to the final doses of 10⁶ particles mL⁻¹ and 10³ particles mL⁻¹, respectively. This approach allowed us to use the same volume of stock solution for each bead size, avoiding differences in the volume added between bead sizes.

**Table S1**. Number of individuals with genera and class in the experiment with their respective number of individuals ingesting the three beads’ sizes at high and low beads concentration. The three replicates per each concentration and the two concentrations (high and low) are pooled together.

| Group | Genus /Class | 1 | | 3 | | 6 | |
| --- | --- | --- | --- | --- | --- | --- | --- |
|  |  | Number of organisms that ingest beads | Total number of organism | Number of organisms that ingest beads | Total number of organism | Number of organisms that ingest beads | Total number of organism |
| Rotifera | *Keratella* | 119 | 279 | 84 | 279 | 97 | 329 |
|  | *Keratella quadrata* | 1 | 5 | 0 | 5 | 4 | 5 |
|  | *Trichocerca* | 33 | 78 | 23 | 61 | 25 | 72 |
|  | *Lecane* | 2 | 17 | 3 | 11 | 7 | 19 |
|  | *Lepadella* | 9 | 14 | 0 | 1 | 3 | 11 |
|  | *Colurella* | 2 | 6 | 1 | 6 | 0 | 7 |
|  | *Sincheta* | 4 | 13 | 3 | 11 | 5 | 17 |
|  | *Cephalodella* | 5 | 7 | 5 | 11 | 2 | 5 |
|  | *Kellicottia* | 0 | 1 | 0 | 0 | 1 | 2 |
|  | *Monommata* | 7 | 9 | 2 | 9 | 5 | 13 |
|  | *Polyarthra* | 0 | 1 | 0 | 0 | 0 | 0 |
|  | *Bdelloids* | 0 | 0 | 0 | 2 | 0 | 1 |
|  | *Trichotria* | 4 | 5 | 0 | 2 | 2 | 6 |
| Cladocera | *Alona* | 0 | 2 | 0 | 0 | 4 | 4 |
|  | *Alonella* | 6 | 6 | 1 | 1 | 1 | 1 |
|  | *Bosmina* | 0 | 1 | 0 | 1 | 0 | 0 |
|  | *Pleuroxus* | 0 | 0 | 1 | 1 | 1 | 1 |
|  | *Ceriodaphnia* | 8 | 9 | 9 | 10 | 9 | 14 |
| Copepoda | *Calanoida* | 6 | 7 | 1 | 2 | 5 | 12 |
|  | *Cyclopoida* | 12 | 25 | 15 | 21 | 15 | 35 |
|  | Nauplii | 16 | 73 | 30 | 74 | 22 | 90 |
| Ostracoda |  | 7 | 8 | 6 | 8 | 6 | 14 |

**Additional Results Information**

In the following tables (S2–S8), n represents the number of observations. The differences in sample size among organisms are due to missing data, as some organisms were not present in the experimental samples.

Table S2. Kruskal-Wallis test for the three sizes of MPs per each group at high and low concentrations of MPs

| Kruskal-Wallis test for the size 1-3-6 | | | | |  |  |  |
| --- | --- | --- | --- | --- | --- | --- | --- |
| Concentration | Group | y. | n | statistic | df | p | method |
| High | Cladocera | Perc | 9 | 2 | 2 | 0.368 | Kruskal-Wallis |
| Low | Cladocera | Perc | 9 | 1.859327 | 2 | 0.395 | Kruskal-Wallis |
| High | Copepoda | Perc | 9 | 2.440678 | 2 | 0.295 | Kruskal-Wallis |
| Low | Copepoda | Perc | 8 | 0.809524 | 2 | 0.667 | Kruskal-Wallis |
| High | Nauplii | Perc | 9 | 2.19209 | 2 | 0.334 | Kruskal-Wallis |
| Low | Nauplii | Perc | 8 | 4.229167 | 2 | 0.121 | Kruskal-Wallis |
| High | Ostracoda | Perc | 8 | 1.666667 | 2 | 0.435 | Kruskal-Wallis |
| Low | Ostracoda | Perc | 5 | 0.666667 | 2 | 0.717 | Kruskal-Wallis |
| High | Rotifera | Perc | 9 | 3.2 | 2 | 0.202 | Kruskal-Wallis |
| Low | Rotifera | Perc | 9 | 1.911504 | 2 | 0.385 | Kruskal-Wallis |

Table S3. Kruskal-Wallis test to test the difference between high and low MPs concentration in each group.

| Kruskal-Wallis test for Concentration | | | |  |  |  |
| --- | --- | --- | --- | --- | --- | --- |
| Group | .y. | n | statistic | df | p | method |
| Cladocera | Perc | 18 | 6.266582 | 1 | **0.0123** | Kruskal-Wallis |
| Copepoda | Perc | 17 | 12.23893 | 1 | **0.000468** | Kruskal-Wallis |
| Nauplii | Perc | 17 | 12.33249 | 1 | **0.000445** | Kruskal-Wallis |
| Ostracoda | Perc | 13 | 9.986364 | 1 | **0.00158** | Kruskal-Wallis |
| Rotifera | Perc | 18 | 12.88254 | 1 | **0.000332** | Kruskal-Wallis |

Table S4. Wilcoxon test with Bonferroni correction for the differences between group at high and low MPs concentrations, the three beads’ sizes are pooled together. Ostracoda are missing from the pairwise comparison as 4 observations were missing.

| Concentration | .y. | group1 | group2 | n1 | n2 | statistic | p | p.adj | p.adj.signif |
| --- | --- | --- | --- | --- | --- | --- | --- | --- | --- |
| High | Perc | Cladocera | Copepoda | 9 | 9 | 64 | 0.026 | 0.154 | ns |
| High | Perc | Cladocera | Nauplii | 9 | 9 | 64 | 0.026 | 0.154 | ns |
| High | Perc | Cladocera | Rotifera | 9 | 9 | 68 | 0.011 | 0.065 | ns |
| High | Perc | Copepoda | Nauplii | 9 | 9 | 59 | 0.11 | 0.66 | ns |
| High | Perc | Copepoda | Rotifera | 9 | 9 | 52.5 | 0.308 | 1 | ns |
| High | Perc | Nauplii | Rotifera | 9 | 9 | 26 | 0.215 | 1 | ns |
| Low | Perc | Cladocera | Copepoda | 9 | 8 | 50.5 | 0.131 | 0.786 | ns |
| Low | Perc | Cladocera | Nauplii | 9 | 8 | 50 | 0.159 | 0.954 | ns |
| Low | Perc | Cladocera | Rotifera | 9 | 9 | 51 | 0.362 | 1 | ns |
| Low | Perc | Copepoda | Nauplii | 8 | 8 | 31 | 0.949 | 1 | ns |
| Low | Perc | Copepoda | Rotifera | 8 | 9 | 27 | 0.375 | 1 | ns |
| Low | Perc | Nauplii | Rotifera | 8 | 9 | 34.5 | 0.919 | 1 | ns |

Table S5. Kruskal-Wallis test to test differences between high and low concentration in *Keratella* spp. and *Trichocerca* spp.

| Group | .y. | n | statistic | df | p | method |
| --- | --- | --- | --- | --- | --- | --- |
| Keratella | Perc | 18 | 13.57393 | 1 | **0.000229** | Kruskal-Wallis |
| Trichocerca | Perc | 18 | 13.60373 | 1 | **0.000226** | Kruskal-Wallis |

Table S6. Kruskal-Wallis test to test differences between keratella and trichocerca at high and low concentration, the size of MPs is pooled together.

| Group | .y. | group1 | group2 | n1 | n2 | statistic | p | p.adj | p.adj.signif |
| --- | --- | --- | --- | --- | --- | --- | --- | --- | --- |
| High | Perc | Keratella | Trichocerca | 9 | 9 | 0.707513 | 0.479248 | 0.479248 | ns |
| Low | Perc | Keratella | Trichocerca | 9 | 9 | 0.242483 | 0.808406 | 0.808406 | ns |

Table S7. Kruskal-Wallis test to test differences between high and low concentration in Calanoida and Cyclopoida

| Group | .y. | n | statistic | df | p | method |
| --- | --- | --- | --- | --- | --- | --- |
| Calanoida | Perc | 12 | 2.957983 | 1 | 0.0855 | Kruskal-Wallis |
| Cyclopoida | Perc | 17 | 13.12601 | 1 | **0.000291** | Kruskal-Wallis |

Table S8. Kruskal-Wallis test to test differences between Calanoida and Cyclopoida at high and low MPs concentrations.

| Concentration | .y. | n | statistic | df | p | method |
| --- | --- | --- | --- | --- | --- | --- |
| High | Perc | 16 | 0.889299 | 1 | 0.346 | Kruskal-Wallis |
| Low | Perc | 13 | 0.216667 | 1 | 0.642 | Kruskal-Wallis |
